# Closing Remarks on the First Special Issue “Acute Care for Traumatic Injuries and Surgical Outcomes”

**DOI:** 10.3390/jcm15030994

**Published:** 2026-01-26

**Authors:** Matthias Fröhlich, Michael Caspers

**Affiliations:** 1 Department of Traumatology, Orthopedic Surgery and Sports Traumatology, Cologne-Merheim Medical Center (CMMC), Witten/Herdecke University, 51109 Cologne, Germany; caspersm@kliniken-koeln.de; 2 Institute for Research in Operative Medicine (IFOM), Cologne Merheim Medical Center (CMMC), University of Witten/Herdecke, Ostmerheimerstr. 200, 51109 Cologne, Germany

Care for traumatic injuries continues to face significant challenges in modern healthcare systems. Despite advances in diagnostics, surgical techniques, and perioperative care, the management of severely injured patients remains highly complex. The demographic shift toward an aging population has led to an increasing number of trauma patients with relevant comorbidities, frailty, and a need for specialized perioperative strategies. This trend complicates both surgical decision making and postoperative recovery.

At the same time, trauma systems must respond to a high variability of injury patterns, ranging from polytrauma after high-energy accidents to fragility fractures in elderly patients. Timely identification of life-threatening injuries and rapid initiation of appropriate interventions remain crucial for survival, yet logistical constraints and resource availability often limit the ability to deliver standardized, high-quality care.

As Guest Editors, we are honored to conclude the first Special Issue on “Acute Care for Traumatic Injuries and Surgical Outcomes” in the Journal of Clinical Medicine. This collection reflects the broad spectrum of challenges in modern trauma care—from emergency resuscitation to surgical decision making and postoperative recovery. Our aim was to gather high-quality contributions that not only address acute clinical questions but also provide perspectives for future research and practice.

**Key Contributions of the Special Issue**

Several studies published in this issue merit particular attention, as they exemplify the diverse and complementary directions in which trauma research is advancing:

Several studies could already demonstrate that the early stabilization of flail chest is associated with improved outcomes [1]. Now, analyzing the “Impact of Surgical Stabilization of Flail Chest Injuries on Postoperative Computed Tomography Lung Volumes” highlights how surgical stabilization of flail chest injuries improves not only chest wall stability but also postoperative lung function, as demonstrated by advanced CT volumetric analysis [2]. This paper emphasizes the importance of functional outcomes in evaluating surgical strategies.

Based on one of the largest trauma databases, the analysis “Epidemiology and Mortality of Surgical Amputations in Severely Injured Patients with Extremity Injuries—A Retrospective Analysis of 32,572 Patients from the TraumaRegister DGU^®^, Chemnitz, Germany” provides robust epidemiological insights into surgical amputations [3]. Its findings are crucial for risk assessment, prognostication, and clinical decision making in patients with devastating extremity injuries.

A measure routinely performed in other clinical situations was investigated in its specific application in trauma patients. By analyzing “The Efficacy of Intraosseous Access for Initial Resuscitation in Patients with Severe Trauma: A Retrospective Multicenter Study in South Korea”, Kim et al. demonstrate in a multicenter setting its effectiveness as a rapid and reliable method, reinforcing its value in situations where time is critical and conventional access is challenging [4].

**Outlook**

Taken together, these studies underscore the multidimensional nature of trauma care. From chest wall stabilization and vascular access to spinal and extremity trauma, the Special Issue demonstrates how innovation, large-scale data, and functional outcome assessment together advance both the science and practice of trauma management.

**Gratitude**

We sincerely thank all contributing authors, reviewers, and the editorial team of the Journal of Clinical Medicine for their dedication. The success of this Special Issue encourages us to continue fostering collaboration across specialties and to further strengthen the evidence base for acute trauma care. We look forward to continuing this work in the forthcoming second edition.
